# Transactions Medical Association of Georgia

**Published:** 1874-09

**Authors:** 


TRANSACTIONS OF THE MEDICAL ASSOCIATION OF GEORGIA, at its Twenty-Fifth Annual Meeting, held in Thomasville, Georgia, April 1st, 2d, and 3d, 1874.

We have received at last from the Secretary the annual volume of Transactions—delayed in its publication by the embarrassed condition of the finances of the Association.

Besides the journal of proceedings, an abstract of which we published on the first of May, the volume contains: the President's address; medication of nursing children through the mother's milk, by Juriah Harriss, M.D.; remarks on the duality of the syphilitic poison, by J. C. LeHardy, M.D.; case of fracture of the patella, followed by bony union, by W. S. Armstrong, M.D.; sympathetic ophthalmia, by A. W. Calhoun, M.D.; dry gangrene of both feet, syphilitic, by B. S. Purse, M.D.; the pine forests of southern Georgia, by T. S. Hopkins, M.D.; gonorrhœal stricture, by R. T. Kendrick, M.D.; report of the practice of medicine in the third district, by T. F. Walker, M.D.; wound and gangrene of arm, amputation at shoulder joint, by T. F. Walker, M.D.; report on surgery of the fourth district, by Carlisle Terry, M.D.; scrotal hernia, strangulation, in patient eighty years old, operation, cure, by G. J. Grimes, M.D.; woman and her peculiarities, by T. J. Word, M.D.; hydrocele cured by one tapping, by T. S. Mitchell, M.D.; subinvolution of the uterus, by V. H. Taliaferro, M.D.; is dysmenorrhœa produced or influenced by the size of the cervical canal? by D. W. Hammond, M.D.; report on surgery of the eighth district, by DeSaussure Ford, M.D.; œsophagotomy, by A. F. Durham, M.D.; cases of urinary calculus treated by L. A. Dugas, M.D.; cases of surgery, by DeSaussure Ford, M.D.; ovarian cyst, tapped and injected, by G. F. Wirsen, M.D.; my experience in the bi-lateral operation for stone, by F. A. Stanford, M.D.; operation for pudendal hernia, by P. M. Tidwell, M.D.; gunshot wound of the scrotum, by Robert P. Myers, M.D.; thirty-seven cases of urinary calculus, by W. F. Westmoreland, M.D.; gunshot wound of the chest, by Paul Faver, M.D.

